# The Intertwining Roads between Psychological Distress and Gut Microbiota in Inflammatory Bowel Disease

**DOI:** 10.3390/microorganisms11092268

**Published:** 2023-09-09

**Authors:** Georgiana-Emmanuela Gîlcă-Blanariu, Cristina Gabriela Șchiopu, Gabriela Ștefănescu, Cătălina Mihai, Smaranda Diaconescu, Vlad Adrian Afrăsânie, Vasile Valeriu Lupu, Ancuța Lupu, Alexandra Boloș, Cristinel Ștefănescu

**Affiliations:** 1 Department of Gastroenterology, University of Medicine and Pharmacy “Grigore T. Popa”, 700115 Iasi, Romania; georgiana.gilca@gmail.com (G.-E.G.-B.); catalinamihai@yahoo.com (C.M.); 2 Department of Psychiatry, University of Medicine and Pharmacy “Grigore T. Popa”, 700115 Iasi, Romania; alex_andra_bolos@yahoo.com (A.B.); cristinel.stefanescu@gmail.com (C.Ș.); 3 Department of Pediatrics, University of Medicine Titu Maiorescu, 040441 Bucharest, Romania; turti23@yahoo.com; 4 Regional Oncology Institute, 700483 Iasi, Romania; vlad_afrasanie@yahoo.com; 5 Department of Pediatrics, University of Medicine and Pharmacy “Grigore T. Popa”, 700115 Iasi, Romania; valeriulupu@yahoo.com (V.V.L.);

**Keywords:** inflammation, inflammatory bowel disease, microbiome, gut-brain axis, psychiatry, anxiety, depression

## Abstract

Inflammatory bowel disease represents one of the most life-altering gastrointestinal pathologies, with its multifactorial nature and unclear physiopathology. The most relevant clinical forms, ulcerative colitis and Crohn’s disease, clinically manifest with mild to severe flares and remission periods that alter the patient’s social, familial and professional integration. The chronic inflammatory activity of the intestinal wall determines severe modifications of the local environment, such as dysbiosis, enteric endocrine, nervous and immune system disruptions and intestinal wall permeability changes. These features are part of the gastrointestinal ecosystem that modulates the bottom-to-top signaling to the central nervous system, leading to a neurobiologic imbalance and clinical affective and/or behavioral symptoms. The gut-brain link is a bidirectional pathway and psychological distress can also affect the central nervous system, which will alter the top-to-bottom regulation, leading to possible functional digestive symptoms and local inflammatory responses. In the middle of this neuro-gastrointestinal system, the microbiome is a key player, as its activities offer basic functional support for both relays. The present article presents current scientific information that links the pathophysiology and clinical aspects of inflammatory bowel disease and psychiatric symptomatology through the complex mechanism of the gut-brain axis and the modulatory effects of the gut microbiota.

## 1. Introduction

The physiology of the gut-brain axis has been a subject of interest for research and clinical observations in the past decade, especially regarding the linkage between gastroenterology and psychiatry. The most notorious argument in favor of gut-brain connection is represented by functional gastrointestinal disorders which manifest with both psychiatric and digestive symptoms. Research on intestinal disorders brought new information that links the intestinal ecosystem with neuro-biologic mechanisms through different pathophysiologic mechanisms [1]. On a clinical scale, these connections are reflected in the association between gastrointestinal symptoms and anxiety, depression, behavioral disruptions, neurodegenerative pathologies and even spectrum disorders. On a microscopic scale, scientific evidence proves that the gut microbiome is the key modulatory factor of the gut-brain axis and its activities involve more than simple metabolic tasks. It appears that inflammation is significantly influenced by gut dysbiosis. Moreover, gut inflammation sets up the pathophysiologic mechanisms that will disrupt the intestinal function and barrier and will trigger the central nervous system response via vagal signaling. In addition, the central nervous system coordinates the intestinal environment through top-bottom signaling, which regulates motility, gut wall permeability and local endocrine, immune and metabolic mechanisms [2].

As the bidirectional communication and balance between the intestinal ecosystem, the microbiome and the brain become more defined in the scientific literature, the most involved pathologic factor between the relays of the axis seems to be the chronic inflammatory response, especially when it affects the intestinal function. As such, inflammatory bowel disease (IBD) has become a research target in the gut-brain axis topic. During active flares of the disease, but also in remission, the life quality of patients is strongly affected, leading to poor social and familial integration, professional disruption and disrupted behavior, which represent vulnerable factors for anxiety and depressive symptoms or sleep impairment in the evolution of the pathology [2,3]. Many IBD patients associate affective and/or behavioral disorders throughout their disease course, but due to the significant somatic symptoms, mental health status has been treated as a secondary factor and not as a comorbidity. Currently, due to the gut-brain axis and microbiome findings, the importance of psychological distress seems to be equally relevant in the IBD physiopathology as the digestive factors [4].

## 2. Materials and Methods

The present article provides an overview of current knowledge linking stress, anxiety, depression and sleep alteration to IBD, cumulating the information available to the present date in the scientific literature and covering clinical and physiological mechanisms involved in the complex connectivity between the gastrointestinal (GI) tract, the gut microbiome and the central nervous system (CNS).

The scientific literature was browsed using the PubMed, ResearchGate and Elsevier databases. Keywords included: gut-brain axis, inflammatory bowel disease, anxiety, depression, microbiome, stress, inflammation, bacterial strain and gut-brain modulation. In addition, terms like “ulcerative colitis” and “Crohn’s disease” were combined with the other search terms for a specific argumentation of the subject. The research accessed online articles and book chapters dating back to 2010 for clinical studies and 2000 for review articles and theoretical information. Over 200 articles were found matching the keywords. Articles that were vague and not focusing on IBD on the gut-brain axis subject or not focusing on specific aspects of the subject were excluded. A number of 124 articles were included in this review. The research included both paid and open access articles, with human and animal studies featuring relevant results for our theme. The research is intended to primarily cover IBD patients with psychiatric symptoms, but, also, the connection between primary affective and behavior disorders that develop IBD symptoms and the bidirectional influence over the clinical evolution of both symptom categories. 

## 3. The Role of Stress, Anxiety and Depression on the Inflammatory Process

As IBD severely affects patients’ quality of life, psychological distress tends to be a part of the clinical manifestations. Still, the increasing evidence about the connection between the CNS and gut microbiota brought a new perspective on the subject. Studies on functional digestive disorders have defined the multiple ways in which the brain affects the intestinal environment, and vice versa, through the microbiome [5]. A big part of the pathophysiology of the gut-brain axis is represented by inflammation, whether acute or chronic, as it activates local and systemic reactions with bidirectional consequences. As such, IBD and psychiatric disorders were the next link in the research field. A very interesting finding from a study published in 2021 offers a great entry in that perspective. Fecal matter transplantation was conducted from IBD patients with and without depression to mice. The mice that received fecal matter from patients with IBD and depression developed a more severe form of IBD-like colitis compared with the animals receiving fecal matter from IBD patients with no depression, but also caused affective behavioral disruptions. The findings also revealed an increased local inflammatory response, with high expression of IL-1β and IL-6, but also an increased systemic and nervous system inflammatory response with high levels of lipopolysaccharide (LPS) and decreased levels of brain-derived neurotrophic factor [6]. These results suggest that inflammation and dysbiosis are tightly linked and are part of the pathologic mechanism that connect the gut environment and the CNS. Moreover, no matter the cause of the dysbiosis, the disruption of normal bacterial strains represents a source of maintaining the inflammatory response, which will transcend from the local environment to the CNS and back, supporting a real vicious cycle [7].

### 3.1. Gut-Brain-Microbiome Axis and Inflammation

The intestinal ecosystem communicates with the CNS through its multiple extra-digestive, anatomic and functional activity. The neurologic activity is formed by bidirectional vagus nerve signaling but also through the activity of local neurotransmitters resulting from bacterial metabolism and of the enteric nervous system. Moreover, the bacterial activity regulates the neuronal activity, such as myelination, microglial maturation and neurogenesis [8].

Another link between the gastrointestinal tract and the brain is represented by the hypothalamic-pituitary-adrenal axis (HPA axis) as local or neurologic distress signals the others relay to react to stimuli. Chronic stress leads to hyperactivity of the HPA axis and, subsequently, to altered intestinal function, but, also, dysbiosis and gut inflammation can lead to HPA axis activation, which manifests with enhanced secretion of cortisol and adrenaline [9].

The metabolic activity of the microbiome has a very important role in the functional mechanisms of the intestinal environment. Metabolites like short-chain fatty acids (SCFAs), resulting from bacterial breakdown of carbohydrates, have important roles like modulation of the inflammatory response with inhibitory effect over cytokines, microglial function (as they pass through the blood-brain barrier) and secretion of gut peptides that act as hormonal precursors (such as serotonin) [10]. Other important peptides are zonulin, which has a great role in the local inflammatory response during dysbiosis [11], and LPS, which are products of bacterial activity, and both can cross into systemic circulation through intestinal wall shafts during permeability disruptions caused by dysbiosis and inflammation [12].

Dysbiosis can alter all functions connecting the gastrointestinal system and the CNS, mostly because the lack of bacterial activity will lead to imbalanced metabolism and pathologic strain overgrowth, which will in turn activate and support the enteric immune system activation and expression of inflammatory cytokines. This mechanism will not only alter the motility, secretion and sensitivity of the intestinal system, but will also trigger altered signaling to the brain that will enhance the digestive function and will affect mood and behavior [13].

Although IBD pathogenesis is not completely elucidated, most of the theories support that an exaggerated immunologic activity is the cause of digestive alterations. This immune activation could have genetic susceptibility and environmental triggering factors, but could also be caused by the overexpression of pathogenic bacteria, dysbiosis and subsequent altered intestinal function [14]. The evolution of both IBD subtypes—Crohn’s disease (CD) and ulcerative colitis (UC)—involves alternations between active structural and functional intestinal injury and remission periods. Extra-digestive symptoms are often represented by anxiety and depressive manifestations or sleep impairment, which will affect the evolution of the pathology [3,15]. The local inflammatory response could alter the CNS function via the gut-brain axis, but it seems that anxiety and depression could be linked to the systemic inflammatory response, as the expression of cytokines has been found in patients with mood disorders. Interestingly, a study conducted on LPS immune-stimulated patients revealed that the inflammatory response to circulating LPS increased was associated with more severe depression and anxiety symptoms compared with the case of basal inflammatory activity. As such, this result brings another argument to the pathophysiologic linkage between intestinal inflammation and mood disorders [16].

### 3.2. Depression, Anxiety and Inflammatory Markers

Independently from the gut-brain axis mechanism, depression, anxiety and stress in general have been linked to systemic inflammatory activity. The studies on this topic are still controversial, as the association between inflammatory markers and anxiety or depression lack quantitative arguments. Several proinflammatory cytokines, tumor necrosis factor (TNF-α) and C-reactive protein (CRP), are the most-studied biomarkers in this regard. Although their presence has been linked to mood and anxiety disorders, the connection between their levels and the severity of psychiatric symptoms remain inconclusive [17]. Chronic or severe stress might trigger depressive and anxiety disorders, but could also activate inflammatory response through the sympathetic and parasympathetic nervous system. At the same time, some of the immune cells express neurotransmitter receptors on their surface for dopamine, serotonin, noradrenaline and endorphins, which makes them responders to signals of the nervous system [18]. 

TNF-α, IFN- α, IL-1, IL-1β and IL-6 are mostly found with increased concentrations in the serum of depression and anxiety patients. In depressive subjects, the low levels of serotonin could be explained by the immune theory, as circulating cytokines could trigger the activity of enzymes related to tryptophan metabolism. These enzymes could further induce a decrease in tryptophan levels through the kynurenine. This chain reaction leads not only to low serotonin levels, but to neurotoxicity alike because of the kynurenine products [19]. 

Anxiety disorders have been researched in many studies alongside depression, but also independently. A systematic review published in 2019 found only 14 studies amongst 1718 in total to be relevant for the theme. Although preliminary evidence revealed a certain degree of inflammatory response in generalized anxiety disorder patients, the pathophysiologic connection between the two remains inconclusive [20].

### 3.3. Inflammation and Oxidative Stress in Mood Disorders and Anxiety

The modern literature brought rich arguments in favor of the association of oxidative stress, neuroinflammation and psychiatric disorders. Brain structure and function depend on high oxygen intake, and they are more sensitive to oxidative stress as their antioxidant activity is less potent. Increased levels of reactive oxygen species with low antioxidant activity will result in the activation and support of inflammation and subsequent cell degradation. In psychiatric disorders, oxidative stress appears to be involved not only in mood and anxiety disorders but also in neurodegenerative pathologies, as the antioxidant activity diminishes with age and it could be a reason for neuronal DNA and structural damage [21]. Also, beyond oxidative-stress-induced neurotoxicity, structural effects and inflammation, reactive oxygen species have the ability to stimulate the HPA axis and to disrupt GABA and serotonin signals. In fact, the interplay between oxidative stress and the HPA axis is somewhat bidirectional, as psychological or physical stress activates glucocorticoid release, which influences the formation of reactive oxygen species in the nervous system through mitochondrial metabolism [22]. Therefore, chronic stress leads to activation of the HPA axis and increased glucocorticoid release, which fail to modulate inflammatory activity due to cortisol-receptor resistance. Chronic inflammation will lead to excessive oxidative stress and low antioxidant defense, but will also stimulate the dysregulated HPA axis and will further support the pathophysiologic cycle of neurotoxicity, structural neural damage, dopaminergic- and serotoninergic-disrupted transmission and will diminish the activity and level of BDNF [23]. Key players in breaking this vicious cycle could involve short-chain fatty acids, which could enhance mitochondrial metabolism during oxidative stress and lower reactive oxygen species formation, but also antidepressants, whose neuroprotectant effect might lower inflammatory activity and, thus, lower oxidative reaction. Still, more clinical data should be required in order to formulate an extensive hypothesis [24,25].

## 4. The Bidirectional Relationship between Gut Dysbiosis, Anxiety and Depression

Inflammatory cytokines, such as IL-1β, TNF- α and IL-6 but also HPA axis imbalanced regulation of glucocorticoids, are mainly present in depression and anxiety patients. Interestingly, the same immune system and endocrine function disruptions appear in IBD and functional gastrointestinal disorders, which are pathologies with associated stress-related, mood and behavioral symptoms. Moreover, oxidative stress’s relation with short-chain fatty acids that are produced by the microbiome brings more arguments in favor of the important connection between the intestinal ecosystem and the CNS [26].

### 4.1. Functional Considerations over Microbiome and Dysbiosis in Psychiatric Disorders

Through pre-clinical studies, researchers were able to find that the gut microbiome plays the central role in modulating the gut-brain axis, as its composition and activity are able to regulate more than digestive functions. Starting from germ-free mouse studies, it has been revealed that dysbiosis is affiliated with mood and behavior disorders with chronic inflammatory states due to microglial disfunction and neurodegenerative activity, especially in young subjects [27]. 

The current state-of-the-art definitions describe the microbiome as an independently ”living organ” with complex functions and roles. It is involved in the metabolism of SCFAs, the balance between fatty acids’ oxidative process and lipogenesis, vitamin synthesis, endocrine functions by modulating neuropeptides and neurotransmitter secretion and immune functions, as it takes part in inflammatory activation with T cells and cytokine activity signaling. Through these functions, the gut bacteria are able to involve themselves in both HPA and vagal signal modulation [28].

Chronic stress, depression, anxiety and sleep disturbances are correlated with the hyperactivity of the HPA axis and immune system. A lack of regulatory feedback over the HPA axis, due to receptor resistance, enables both glucocorticoid and inflammatory marker levels to rise, leading to oxidative stress. On the other hand, dysbiosis and pathologic strains may trigger enteric inflammatory responses due to the dysregulation of local functions. The presence of pathologic strains activates inflammatory markers and activity; a lack of beneficial strains may lead to decreases in tryptophan and SCFA metabolism, with a direct influence the integrity of the intestinal wall and neurotransmitter activity, but also will allow for further production of reactive oxygen species and lipopolysaccharides, which enhance local, systemic and neuronal toxicity [29]. SCFAs represent a fermentation product of anaerobic bacteria. SCFAs modulate the secretion of neurotransmitters like glutamate, GABA and the expression of tryptophan by stimulating the apical membrane receptors. This implies a bottom-to-top regulatory effect in the gut-brain axis. Also, a CNS neurotransmitter disruption, due to psycho-social stress, for example, will signal altered information to the gut, influencing the microbiome’s composition. This is part of the pathophysiologic mechanism involved in anxiety and depression [30].

Dysbiosis and gut populations with pathologic strains, especially Gram-negative bacteria, will stimulate the production of lipopolysaccharides (LPS), which are immunogenic enzymes. On a local scale, LPS lead to inflammatory responses and hyperactivation of the HPA axis with increasing levels of cortisol. Glucocorticoids lower their own secretion via negative feedback, but in anxiety and depressive patients, there is a level of cortisol-resistant receptors, which would explain the pathologic loop of keep both inflammatory markers and cortisol levels up [31]. A lack of beneficial strains, specialized in SCFA metabolism, could cause not only inflammatory responses and neurotransmitter disturbances, but, indirectly, could support oxidative stress and its toxic products. The capacity of blocking reactive oxygen species formation of SCFAs, especially butyrate and acetate, which are mainly produced by gut bacteria fermentation processes, is suspected to have not only neuropsychiatric benefits but they could also be a future therapeutic target in other pathologies, as the antioxidative and anti-inflammatory effect might have general organic benefits, especially in LPS-induced pathophysiologic mechanisms [32,33,34]. Butyrate seems to exert anti-inflammatory properties, and SCFA-producing bacteria could even have a specific role in immune regulation between the gut and the brain. It seems that the anti-inflammatory role of butyrate is given by its inhibitory effect over nuclear factor κB (NF-κB) which activates IL-1β, IL-2, IL-6, TNF-α, nitric oxide synthesis, vascular adhesion molecules and cyclooxygenase-2. Also, NF-κB seems to be constantly active in colorectal cancer and IBD. The complex role of SCFA-producing bacteria brings yet another argument in favor of gut-brain axis pathophysiology [35].

### 4.2. The Gut Barrier and the Blood-Brain Barrier

The integrity of the intestinal wall represents a primary mechanical defense mechanism against toxic metabolites, pathologic bacteria and inflammatory markers entering the circulatory system and reaching the CNS. The danger of those substances and infectious agents entering the CNS lies in the blood-brain barrier. Both the blood-brain barrier and the intestinal wall barrier share similar structural and functional elements such as lymphatic and blood vessels, epithelial cells, endothelial tissue, macrophagic activity and cellular tight junctions [36]. Those tight junctions are represented by specific proteins that form the cytoskeleton of the intestinal wall by binding to the actin base. Occludin, claudins, junctional adhesion molecules and zonulin are the most important proteins of this type. SCFA metabolites regulate the expression of tight junction proteins in the intestinal wall, which makes gut-wall permeability directly dependent on the SCFA-producing microbiome [37].

The intestinal barrier is not only a mechanical wall, but also a functional one, as its permeability is dependent on microbiota composition with bacterial strains that produce SCFAs which regulate the expression of these tight junction proteins. The tight junctions of the intestinal wall enclose the functional barrier in order to keep all possible toxic and infectious agents away from the system [38]. 

**Figure 1:**
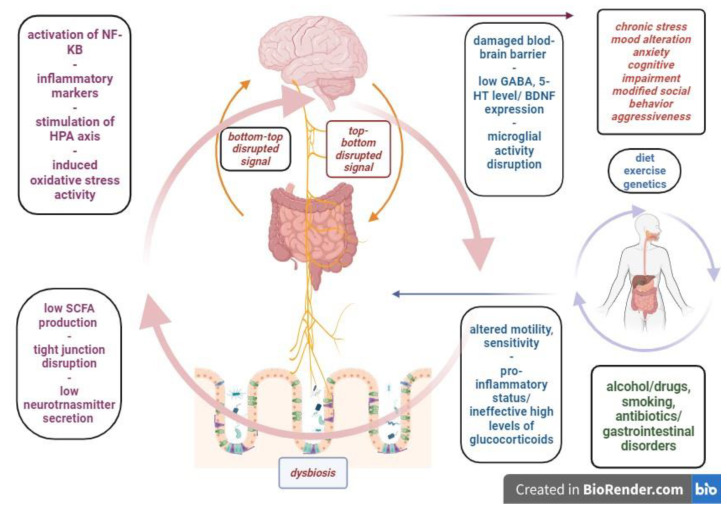
The pathophysiologic interplay between intrinsic and extrinsic factors and mechanisms within the microbiome-gut-brain axis.

Dysbiosis will alter gut permeability by depleting SCFAs and, subsequently, the expression of tight junctions. Local inflammatory responses will trigger the production of cytokines; TNF-α and pathogenic bacteria will produce LPS and other toxic by-products. Also, the rate of reactive oxygen species will increase due to chronic inflammatory activity. As such, the antimicrobial substances are exhausted, the mucin layers are destroyed and, as inflammation advances, the epithelial cells are also damaged from both cytokines and TNF-α, but also from oxidative stress, leading to breakage in the tight junctions of the gut barrier and increased blood flow. This will stimulate the release of toxins, inflammatory markers, microbial metabolites, infectious pathogens and reactive oxygen species into systemic circulation [39]. Many of these substances, such as inflammatory markers, toxic bacterial metabolites and free radicals, are able to pass through the blood-brain barrier, altering its permeability and leading to functional and structural alterations in the CNS. A recent and interesting study shows a remarkable association between plasma levels of permeability biomarkers of the gut and blood-brain barrier connected to depression and anxiety symptom severity scores. This kind of study could further be expanded to other mechanisms that could offer more details of the pathophysiological aspects of the gut-microbiome-brain axis [40]. Neuroinflammation and the disruption of neurotransmitter signaling is part of the connection between gut permeability and neuropsychiatric disorders (Figure 1).

Psychological stress, anxiety and depression may cause blood-brain barrier disfunctions through raised inflammatory markers and glucocorticoid levels. Such reactions may release these compounds into the blood stream. Also, disfunctions in the CND may trigger altered signals through the neuro-endocrine communication between the brain and gastrointestinal tract. The combined disruptions will cause modifications to gut motility, microbiome composition and gut barrier permeability in a top-bottom regulatory influence [41,42]. All these mechanisms support the microbiome’s modulatory role within the gut-brain axis with both digestive and neuropsychiatric alterations.

### 4.3. Therapeutic Arguments Linking Dysbiosis, Anxiety and Depression

The gut microbiome is not only an entity dependent on intrinsic physiology and genetic factors, but is also dependent on environmental factors such as diet, lifestyle, exercise and medical conditions that require pharmacologic therapy. The bottom-up regulatory effect of microbial strain variety has been the subject of many preclinical and clinical research projects, all starting from antibiotic exposure and the effects of antibiotic-induced dysbiosis on mood and behavior. The depletion of beneficial gut bacterial strains resulted in increased depressive, anxiety and cognitive symptoms even in subjects with no prior psychiatric manifestations. That could be explained by the fact that dysbiosis enables the pathophysiological mechanism that increases inflammatory activity and oxidative stress, decreases barrier and local defense mechanisms and alters the neuro-endocrine signals alongside barrier function disruptions. Animal model studies have revealed that antibiotic treatment has a positive correlation with anxiety symptoms, depressive symptoms, behavioral changes and cognitive impairments [43]. Also, germ-free animal model studies have brough insights for the pathogenic mechanisms involved in the gut-brain-microbiome interplay and pertinent observations of the beneficial role of the gut microbiome. Germ-free intestinal environments permit a specific microbiota population in order to observe accurate improvements in both digestive and neuropsychiatric manifestations [44].

Most actual studies and systematic reviews have revealed positive correlations between anxiety and depression symptom improvement and probiotic therapy, although there are still few clinical studies [45,46]. More research on specific strains as an accurate linkage between bacterial strains and mood and behavioral manifestations is needed (Table 1).

A Mediterranean diet, vitamin D, B-group vitamins, minerals like magnesium and prebiotics such as inulin and oligosaccharides have been stated to enhance the beneficial role of probiotics, leading to long-term beneficial effects for both digestive symptoms and psychiatric disorders. The overall inflammatory cycle diminished alongside cortisol levels and oxidative stress rates, with depression and anxiety scale improvement and reductions in digestive symptoms. The complex intervention is based on the fact that probiotic supplementation alone could fail to stabilize the enteric system, and for bacterial strains to survive they need prebiotics, dietary fibers, vitamins and minerals in order for them to replicate and properly populate the gastrointestinal tract. On the other hand, El Dib et al.’s systematic review found that probiotics might improve affective and anxiety symptoms, but larger samples and sustained follow-up of the patients is needed in order to correctly assess therapeutic management of the gut-brain axis [57,58]. Magnesium supplementation is a newly emerged probiotic adjuvant, especially when combined with orotic acid. Although small group studies are available, their results seem promising. Combined with probiotics, magnesium orotate seem to improve severe depression symptoms, anxiety manifestation and have an overall anti-inflammatory enteric response. Still, stopping the treatment may lead to symptom relapse, which should be taken into consideration in future studies [59].

The emerging research in the field of the gut-brain-microbiome axis has taken into consideration the usage of the term ”psycho-biotics” in light of using specific bacterial strains with the capacity to modulate and influence mood, behavior, anxiety and maybe even cognitive manifestations apart from their digestive effects. The term psycho-biotic does not only cover probiotics, but also their adjuvant therapies, such as prebiotics and dietary supplements, that are required for a therapeutic result [60]. 

Probiotic supplementation may induce anti-inflammatory responses and lower the oxidative reaction. A study published in 2022 measured the correlation between oxidative stress biomarkers and bipolar disorder scale measurement, with results showing an inverted proportionality between plasma biomarkers of oxidative stress and symptom severity, especially with mood disruption, after probiotic therapy [61,62]. 

## 5. The Relationship between Gut Microbiota and Intestinal Inflammation

There is a significant interplay between the gut and the intestinal microbiota and there are several factors which might be contributing to the dysregulation of this interaction, ranging from genetics to lifestyle, diet and environmental factors, with particular aspects for intestinal inflammation in IBD [63]. 

Not only the presence of various types of microorganisms in the gut is relevant in the context of IBD, but also the interaction between the gut microbiota and the host, considering the role of microbial metabolites in sustaining the maturation and functioning of the host’s immune system, contributing to host’s homeostasis [64]. It has been highlighted that several metabolites derived from the gut microbiota play key roles in mediating the interaction between the microbiota and host. Among these, the dysregulation of bile acid metabolism, the reduction in the level of short-chain fatty acids (SCFAs) and medium-chain fatty acids and changes in the level of sphingolipids and polyamines have been identified as important gut microbiota-derived metabolites changes in various untargeted, heterogenous fecal metabolomic studies [65,66,67,68]. One of the largest and most detailed study, a cross-sectional cohort, including 155 subjects from the PRISM IBD cohort in the USA, identified, using metagenomic sequencing and liquid chromatography tandem mass spectrometry, a difference in the abundance of various gut microbiota-derived metabolites among IBD patients, especially for Crohn’s disease patients, compared with non-IBD subjects, providing insight into the perturbations of the microbiome-metabolome interface in IBD [69]. Furthermore, tryptophan metabolism is dysregulated in IBD, with an inverse correlation between disease activity and tryptophan level [70]. Several results suggest that the increased tryptophan metabolism in this setting is mediated through the kynurenine pathway, with one study identifying the link between tryptophan metabolism and IBD in a large clinical cohort [71]; there is also evidence that the expression of aryl hydrocarbon receptors is reduced in the context of mucosal inflammation in Crohn’s disease patients [72]. Existing data support the involvement of a variety of gut microbial-derived factors together with an abnormal immune response and impaired integrity of the intestinal barrier in contributing to altered intestinal inflammatory responses [73]. 

The genetic components of several immune key players involved in the inflammatory process have been studied through genome-wide association studies, and candidate gene networks impacting host-microbiota interaction have been identified, such as Toll-like receptor (TLR) and nucleotide-binding oligomerization domain (NOD)-like receptor signaling [74]. The alteration of the intestinal barrier in IBD contributes to the disruption of the local homeostasis, increasing the exposure of the host immune system to the luminal microbiota [64]. Several genes, such as ATG16L1, KCNN4 and XBP1, have been linked to the susceptibility to develop IBD, since they code for proteins involved in various cell functions in the intestinal epithelial cells [75]. Preclinical studies have emphasized that the increase in the TNF-α-TNFR2 signaling pathway in intestinal epithelial cells leads to increased expression of myosin light chain kinase, significantly contributing to altering the assembly of tight junctions, therefore reducing the integrity of the intestinal barrier via proinflammatory signals [76]. Moreover, the expression of antimicrobial peptides is decreased in IBD, with evidence that mutations in the CARD15 gene—which encodes the NOD2 protein—were associated with reduced α-defensin production in pediatric patients with IBD with ileal involvement [77,78]. Furthermore, the presence of ATG16L1—which is among the CD risk alleles—predisposes one to altered function of Paneth cells, namely to the loss of the ability to form normal intracellular granules and the consequent decreased production of antimicrobial peptides [79,80]. Consequently, the defects in the epithelial cell barrier favor chronic exposure to both bacteria and bacteria-derived products, contributing to the inflammatory process [81]. 

Both the diversity and composition of gut microbiota have a high impact on the abundance of Th17 cells, which are involved in antibacterial defense, although their excessive activation can contribute to autoimmune inflammation in the gut [82,83]. It has been highlighted in both preclinical studies, in mouse model studies and translated to clinical studies including IBD patients, that Th17 cells exhibit functional plasticity towards Th1 lineage, in a manner dependent on the presence of interleukin- 12 and interleukin-23 secreted by antigen-presented cells, triggered by bacterial-derived signaling and dependent on IFN- γ production [84]. On the other hand, there is a reported paradoxical effect of anti-IL-17 pathway agents, namely Secukinumab, in triggering IBD onset [85,86]. This fact may be explained by the protective role of IL-17 against inflammation by inhibiting Th1 response and maintaining the integrity of intestinal epithelial barrier [87] The pivotal role of Treg cells in IBD pathogenesis is supported by studies on murine models, showing that mice lacking Treg cells develop spontaneous colitis [60]. Forkhead box P3 (FOXP3) is a significant transcriptional regulator involved in the development and function of CD4+ regulatory T (Treg) cells; an increased number of Foxp3+Tregs and high levels of their signature cytokines TGF-β and IL-10 have been identified in the inflamed intestinal wall of IBD patients and also highlighted in a murine model with colitis [88,89]. The strong interrelationship between Treg cells and microbiota is supported by the evidence that Gram-positive commensal bacteria play a prominent role in maintaining Treg homeostasis, as highlighted by experiments where the reconstitution of germ-free mice with Gram-positive spore-forming microorganisms restored the Treg population [90,91]. Another key microbiota player influencing mucosal immunity is *F. prausnitzii*, with studies showing that it alters dendritic cells to modulate the function of a subset of IL-10-producing T cells in order to express molecules such as IL-10, CD39, indoleamine 23-dioxygenase 1 and programmed death-ligand 1 (PDL-1), all of which are important type 1 regulatory T (Tr1)/Treg polarizing molecules [92].

Not only do bacterial components influence the mucosal immune response, but fungi also have an important role, considering that *Candida albicans* can influence the mucosal innate immune cells, through dectin-1-associated pathways in macrophages and TLR4 in neutrophils [93,94].

## 6. Psychological Distress among IBD Patients

Psychological well-being is pivotal for maintaining a healthy status; several symptoms arising during organic diseases have a strong impact on psychological status, which in turn influences the evolution of symptoms. This bidirectional link has been highlighted regarding the place of psychological comorbidity in patients with IBD [95,96].

Among the first systematic reviews investigating a potential link between psychiatric comorbidity and ulcerative colitis, published in 1990, including 138 clinical studies published up to that moment concluded there was no association between psychiatric comorbidity and ulcerative colitis, but most of the studies supporting this conclusion had major methodological deficiencies [97]. Studies conducted afterwards, including prospective case-control studies with large numbers of patients demonstrated an increased prevalence of depression and anxiety disorders among IBD patients, significantly affecting this patient category [98,99,100]. Taking into account that about one in five IBD patients have an associated psychological comorbidity and that undiagnosed anxiety is common in this patient category, even during remission, psychological status evaluations should be included in the multidisciplinary approach of IBD patients [101,102]. Although the increased risk of psychological distress during disease flare is acknowledged, explaining the presence of psychological symptoms during remission has several missing links. Considering that psychological distress leads to somatic effects, the relationship between psychological comorbidity and inflammation has a complex pathophysiological background, relying on the brain-gut axis. Psychological impairment is involved in a stress response through the hypothalamic-pituitary-cortico-suprarenal axis, leading to increased intestinal permeability, but this link has been proven to be bidirectional [103,104]. Furthermore, the influence of psychological distress can be exerted via cathecolamine release during stress responses, leading to mastocyte activation within the digestive tract [105]. 

Taking into account that a proper circadian rhythm is essential in regulating the immune and neuroendocrine systems, it is easy to understand that the presence of fatigue and daytime sleepiness are significant symptoms of various inflammatory diseases. There is a strong link between sleep and appropriate function of the immune system, demonstrated through studies certifying that adequate sleep contributes to consolidating adequate immune responses; studies highlight the link between sleep alteration and altered release of various proinflammatory mediators such as IL-1 and TNF-α [106,107]. Studies on animal models have found that increased IL-1 and TNF-α were associated with increased duration of NREM sleep, particularly of the SWS subtype, while clinical studies pointed to the contribution of IL-6—one of the immune mediators involved in Crohn’s disease pathogenesis—to the reduction in REM phase sleep, promoting the waking state in IBD patients [108]. Moreover, the bidirectional link between depression and altered sleep has raised interest in IBD for clinical research with clinical evidence that anxiety and disease activity are predictive factors for altering the quality of sleep in IBD patients [109,110]. Other data deriving from an observational study including pediatric IBD patients with depression identified through multivariate modeling that qualitative measures of sleep impairment (reflected through daytime dysfunction, decreased sleep quality subjectively identified by the patient and increased sleep latency) were predicted by anxiety and the presence of active disease, but not to biochemical markers of inflammation (evaluated through CRP) [111]. A large Canadian cohort study identified an increased prevalence of anxiety and depression among IBD patients, affecting up to one-third of patients [4], while a Swiss cohort study with a follow-up period of 10 years identified a significant correlation between the presence of depression and the recurrence of IBD symptoms, both for Crohn’s disease (*p* = 0.0007) and ulcerative colitis patients (*p* = 0.0050), and also between anxiety and the recurrence of IBD symptoms. (*p* = 0.031) [99]. The same study highlighted that anxiety had a higher prevalence (17.9%) than depression (10.5%), while the impact of depression on IBD activity was more significant. Considering the pathophysiological aspects, anxiety and depression often coexist and, in addition to stress, augment the inflammatory response [112].

The presence of anxiety and/or depression could also contribute to lower adherence to therapy, as demonstrated by a study conducted by Gray et al. using the Medication Adherence Measure instrument, identifying a 12% decrease in adherence to IBD therapy [113,114]. Moreover, a previous systematic review and meta-analysis revealed moderate evidence of higher anxiety and depression rates among IBD patients compared with healthy controls, with higher rates among patients with active disease compared with remission [115]. There is a potential of developing psychological distress during IBD; among the risk factors for developing psychological disorders in IBD, disease activity and aggressive disease behavior play a central role. However, there are detrimental consequences of psychological impairment beyond those directly related to disease activity, including reduced adherence to therapy and increased request of medical investigation beyond the required regular monitoring [7]. In this setting, the management of psychological comorbidity could contribute to improving disease outcome; one retrospective case-matched study including IBD patients with associated mood disorders receiving antidepressant treatment experienced a reduced number of disease flares, decreased use of corticotherapy and lower addressability for supplementary investigations in the 1-year follow-up period [116]. 

All these findings highlight the importance of screening for psychological disorders among IBD patients for the early detection and management of this psychological impairment in order to optimize the disease outcome.

## 7. The Association of Alterations in Gut Microbiota with Impaired Psychological Status in Patients with Inflammatory Bowel Diseases

Current evidence supports the role of the microbiota-gut-brain axis as a pillar in the link between dysbiosis and alterations of the psychological status. Since there is strong evidence of the presence of dysbiosis in the context of intestinal inflammation and several studies highlight the significant prevalence of psychological distress among IBD patients, it is reasonable to infer that IBD-associated dysbiosis, which contributes to the inflammatory process in the gut, can influence psychological status. The increased intestinal permeability leads to an abnormal enteric nervous system response, contributing to visceral hypersensitivity, although this mechanism has not been clearly stated in IBD. The activation of the enteric nervous system could be possible in this setting, via the exposure to lipopolysaccharides of the local gut bacteria, leading to subsequent afferent nerve stimulation and activation of the brain-gut axis [117].

The complex mechanisms supporting the impact of the IBD-associated dysbiosis on psychological status remain to be elucidated. As prior data suggest, the link between the gut microbiota and the brain is mediated through the hypothalamic-pituitary-adrenal axis, activation of the immune system, the inflammatory response system and oxidative and nitrosative stress and tryptophan catabolites [118,119]. Moreover, recent data support a new mechanism linking tryptophan metabolism to the intestinal inflammation in IBD, even highlighting that modulating the endogenous tryptophan metabolism through reestablishing xanthurenic and kynurenic acids has protective effects, contributing to rewiring of the energy metabolism in intestinal epithelial cells and CD4+ T cells [120]. 

Regarding how to connect the knowledge on mechanisms mediating the relationship between the gut microbiome and altered psychological status in IBD, the data are still scarce. 

A study focusing on the interactions between the composition of the intestinal microbiota (analyzed using 16S rRNA high-throughput sequencing) and psychological outcomes in patients with IBD identified associations between psychological distress and decreases in operational taxonomic units of the *Lachnospiraceae*, *Fusobacteriaceae*, *Ruminococcaceae*, *Veillonellaceae*, *Alcaligenaceae*, *Desulfovibrionaceae* and *Bacteroidaceae* families. The relative abundance of some bacterial genera were correlated with depression, namely *Bifidobacterium*, in patients with Crohn’s disease and *Desulfovibrio* in patients with ulcerative colitis [121]. Further steps have been taken to understand the influence of gut dysbiosis on psychological status in IBD, through studying not only the structural, but also the functional characteristics of microbiota on the severity of depressive symptoms in patients with active IBD. From this study, using the metagenomic microbiota profiles of IBD patients, there is evidence of association for three genera (*Odoribacter*, *Anaerotruncus* and *Alistipes*) and three functional modules (pectin, glycosaminoglycan and central carbohydrate metabolism) with regard to depression among IBD patients [122]. A recent study using polygenic risk scores (PRS) examined the interactions between IBD and the gut microbiome and evaluated the effects of these interactions on the risk for depression. This study brings emerging evidence on the association between interactions of the gut microbiome on IBD and depression, identifying several significant gut microbiome and IBD interactions, such as *Dialister* and CD, *Anaerostipes* and CD, *Alloprevotella* and UC, *Veillonellaceae* and UC and *Lachnospiraceae* in UC and CD. The study also underlines a different risk of depression across sex and age based on the effect of the gut microbiome PRS and IBD PRS interactions. Moreover, the same study identified several candidate genes for depression phenotypes, such as GPM6A, HDAC7, VDR and QRICH1 [123]. Among these genes, GPM6A is already known to be involved in promoting the formation of synapses, with evidence of involvement in brain signaling pathways of psychiatric disorders such as depression [111]. HDAC7 is a histone deacetylase involved in the macrophage-mediated inflammatory response, leading to increasing IL-1β and Ccl2 levels, which are inflammatory mediators, while the vitamin D receptor plays a role in regulating T cells and has been shown to modulate the gut microbiota-host interaction [124]. 

## 8. Conclusions

Anxiety and depression, as well as cognitive impairment and sleep alteration, may be connected to an inflammatory response, a disrupted HPA axis stimulation and oxidative stress. These mechanisms influence one another, maintaining the pathophysiologic cycle.

Independently of the inflammatory background of mood and anxiety disorders, the gut-brain axis has a more complex role in neuropsychiatric disorders. The bidirectional coordination is modulated by the gut microbiota, which influences both local enteric activity and brain signaling though its diverse roles: SCFA metabolism with neurotransmitter expression, modulating inflammatory responses, neuroendocrine signaling and acting as a first line defense of the gut barrier integrity. The gastrointestinal tract and the CNS influence each other in a top-bottom/bottom-top regulation system. Psychosocial stress, depressive states and anxiety manifestation trigger neurobiological responses in the CNS, with subsequent inflammatory responses, oxidative reactions and raised inefficient glucocorticoid levels. This mechanism is not only reflected in clinical mood and behavioral manifestations, but also affects the gut environment by transmitting disrupted signals that will affect bacterial diversity, metabolism, motility, visceral sensitivity and will modify enteric endocrine and immune activity. At the other end of the relay, dysbiosis may lead to failed SCFA metabolism with decreased neurotransmitter secretion, lipopolysaccharide production, consistent inflammatory responses and reactive oxygen species production. This state inversely triggers the CNS, affecting not only the digestive function but also neuronal function and neuro-bio-chemical activity in the brain via sympathetic stimulation, HPA axis stimulation and, most importantly, by increasing gut barrier permeability and realizing toxic metabolites and antigens in the systemic circulation that may pass the blood-brain barrier and directly impact the nervous system. 

Improving the understanding of gut microbiota alterations among IBD patients and their involvement in altering psychological status could contribute to disentangling several pathogenetic mechanisms and could broaden the approach to the treatment of this patient category.

## Figures and Tables

**Table 1:** Specific bacterial strains in research for potential IBD/anxiety, mood and cognitive disorders.

Bacteria Species	Digestive Effects	Psychiatric Effects	Source
*Lactobacillus reuteri* (mice)	Decreased inflammatory activity in colitis	Improvement in anxiety behavior Improvement in depressive symptoms	[47]
*Lactobacillus* *mucosae* *Bifidobacterium longum* (mice)	Synergic effect—inhibition of NF-κB ^1^, TNF-α ^2^—anti-inflammatory effect	Anxiety/depression symptom improvement; BDNF ^3^ expression	[48]
*Lactobacillus plantarum* (mice)	Anti-inflammatory effect	BDNF and 5-HT ^4^ expression Anxiety/depression improvement	[49]
*Lactobacillus casei* (mice)	Anti-inflammatory response, oxidative stress decrease	Mood/behavior/anxiety improvement BDNF expression	[50]
*Lactococcus lactis* subsp. *cremoris* (mice)	Modulation of inflammatory markers and oxidative stress	Cognitive and mood improvement	[51]
*Bacillus subtilis* (human)	Motility, pain improvement Anti-inflammatory effect	Depression symptom improvement	[52]
*Saccharomyces boulardii* (mice)	Anti-inflammatory effect, oxidative stress reduction; gut barrier permeability improvement—histological improvement (LPS ^5^-induced anxiety)	5-HT expression Anxiety symptom improvement	[53]
*Lactiplanbacillus plantarum* (mice)	Increased SCFA ^6^ in induced ulcerative colitis; Gut barrier permeability modulation; Histological improvement;	5-HT expression; mood improvement;	[54]
Biotop^®^ (*Lactobacillus acidophilus*, *Clostridium butyricum*, *Bacillus mesentericus*, *Streptococcus faecalis*) (human)	Improvement in IBS ^7^ symptoms of endoscopic remission patients	Social function improvement * L. acidophilus improves depression severity scores in different study with *L. casei* and *B.bifidum*	[55,56]

^1^ Nuclear factor kappa light chain enhancer of activated B cells. ^2^ Tumor necrosis factor alpha. ^3^ Brain-derived neurotrophic factor. ^4^ 5-hydroxytryptamine. ^5^ Lipopolysaccharide. ^6^ Short-chain fatty acids. ^7^ Inflammatory bowel disease.

## Data Availability

Not applicable.
